# Survival and adaptative strategies of Enterotoxigenic *E. coli* (ETEC) to the freshwater environment

**DOI:** 10.21203/rs.3.rs-6252921/v1

**Published:** 2025-03-19

**Authors:** Åsa Sjöling, Eswari Ashokkumar, Caroline Bjurnemark, Kaisa Thorell, Xue Xiao, Astrid von Mentzer, Yue O. O. Hu, Baoli Zhu, Enrique Joffré

**Affiliations:** Dept Chemistry and Molecular Biology, University of Gothenburg, Gothenburg, Sweden; Department of Microbiology, Tumor and Cell Biology, Karolinska Institutet, Stockholm, Biomedicum A8, Stockholm, Sweden; Department of Microbiology, Tumor and Cell Biology, Karolinska Institutet, Stockholm, Biomedicum A8, Stockholm, Sweden; Dept Chemistry and Molecular Biology, University of Gothenburg, Gothenburg, Sweden; CAS Key Laboratory of Pathogenic Microbiology & Immunology, Institute of Microbiology, Chinese Academy of Sciences, No. 1 West Beichen Road, Chaoyang District, Beijing 100101, PR China; Department of Microbiology and Immunology, Sahlgrenska Academy, University of Gothenburg, Sweden; Department of Microbiology, Tumor and Cell Biology, Karolinska Institutet, Stockholm, Biomedicum A8, Stockholm, Sweden; CAS Key Laboratory of Pathogenic Microbiology & Immunology, Institute of Microbiology, Chinese Academy of Sciences, No. 1 West Beichen Road, Chaoyang District, Beijing 100101, PR China; Department of Microbiology, Tumor and Cell Biology, Karolinska Institutet, Stockholm, Biomedicum A8, Stockholm, Sweden

**Keywords:** Enterotoxigenic E. coli, microbial pollution, freshwater, survival and adaptative strategies, colistin resistance, temporal gene expression, freshwater microcosms, biofilm production

## Abstract

Waterborne pathogenic enterobacteria are adapted for infection of human hosts but can also survive for long periods in water environments. To understand how the human pathogen enterotoxigenic *Escherichia coli* (ETEC) adapts to acute and long-term hypo-osmotic stress and oligotrophic water conditions, this study aimed to explore the effects of short- and long-term freshwater exposure on ETEC isolates by examining transcriptional responses, survival mechanisms, and antibiotic resistance development. RNA sequencing revealed that over 1,700 genes were differentially expressed, with significant transcriptional reprogramming occurring early within the first two hours of water exposure. Early responses included activation of catabolic pathways for nitrogen and carbon assimilation and downregulation of energy metabolism and anabolic processes to mitigate osmotic stress. Notably, the *arnBCADTEF* operon was upregulated, facilitating lipid A modification and membrane enforcement which also confers colistin tolerance. ETEC carries virulence genes on large plasmids which cause diarrheal disease in humans. Plasmid gene analysis indicated repression of virulence genes and upregulation of mobilization and toxin-antitoxin systems during the first 48 hours in water, suggesting a shift towards genetic adaptability. Prolonged exposure over weeks enhanced biofilm formation capacity and adherence to human epithelial cells, and ETEC isolates evolved towards increased colistin resistance. These findings stress the significant influence of freshwater on ETEC adaptive strategies, suggesting a role of waterborne transmission for human pathogens in development of persistence, biofilm formation capability and the emergence of antibiotic tolerance.

## INTRODUCTION

Enterotoxigenic *Escherichia coli* (ETEC) is a major pathogen responsible for moderate-to-severe diarrhea in children under five years old, particularly in low- and middle-income countries (1). It is also a leading cause of travelers’ diarrhea and sporadic food- and waterborne outbreaks (2). ETEC infections are primarily acquired through the consumption of contaminated water and food via the fecal-oral route, posing a significant risk in regions with inadequate water, sanitation, and hygiene (WASH) infrastructure. Globally, at least 1.7 billion people rely on unsafe drinking water sources (3), resulting in approximately one million deaths annually from diarrhea caused by contaminated drinking water (4).

Polluted water serves as a reservoir for ETEC (5, 6), and in endemic regions, ETEC infections tend to increase during warmer, rainy seasons when water quality deteriorates (7). ETEC can persist and survive in various environments, including freshwater and seawater, for extended periods (8, 9). In oligotrophic water environments, ETEC remains viable and infectious by entering a dormant state, a phenomenon also observed in other enteropathogenic bacteria such as *Vibrio cholerae* and *Campylobacter jejuni* (9). This underscores the role of contaminated water as a primary transmission route for ETEC.

ETEC colonizes the human small intestine by means of colonization factors (CFs) which facilitate adherence to the intestinal epithelium, adherence is followed by secretion of heat-labile (LT) and/or heat-stable toxins (ST) that cause the disease symptoms (10). Virulence factor expression in host environments has been extensively studied, revealing that ETEC senses environmental cues such as oxygen, bile, pH, nutrients, and osmolarity to coordinately regulate toxin expression, colonization, and biofilm formation (11–18). However, how ETEC responds and adapts to environmental conditions outside the host remains poorly understood.

In earlier studies of ETEC in freshwater and seawater microcosms we demonstrated that clinical isolates could be cultured for up to three months with detectable virulence and metabolic gene expression while maintaining bacterial cell integrity (8). A more recent study employing transposon mutagenesis and RNA-seq expression profiling of ETEC strain H10407 in water revealed alterations in gene expression related to lipopolysaccharide (LPS) and carbohydrate modifications, as well as reductions in genes associated with cell division and virulence (18). However, comprehensive analyses of the temporal transcriptomic responses and adaptive mechanisms of ETEC during prolonged freshwater exposure remain limited.

Herein, we comprehensively studied the temporal transcriptomic responses of a clinical isolate, ETEC E2265 (CS5 + CS6 LT/STh), and the prototype ETEC H10407 (CFA/I LT, STh/STp) during the transition from a nutritionally rich environment (Luria-Bertani broth, LB) to a nutrient-poor freshwater environment. We evaluated the impact of long-term freshwater incubation on biofilm formation, antibiotic resistance, and adherence to human cells. This work enhances our understanding of conserved and strain-specific adaptive strategies of ETEC in response to short- and long-term exposure to freshwater, highlighting the mechanisms that enable survival and adaptation.

## RESULTS

### ETEC survive with intact transcriptional activity and retained plasmids for extended periods of incubation in freshwater

**Figure 1:**
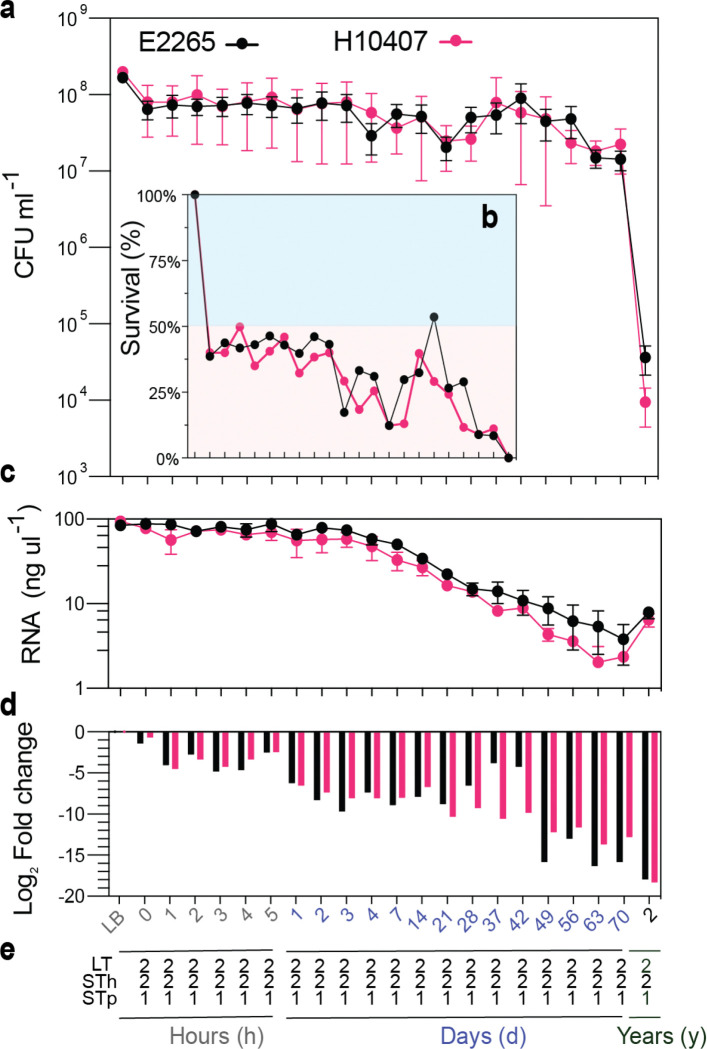
Short- and long-term impact of freshwater exposure on ETEC isolates. **A)** Bacterial viability and **b)** survival (%) in freshwater microcosms. Strains E2265 and H10407 were transferred from LB medium into sterile freshwater microcosms. Water samples for colony-forming unit (CFU/mL) determination were collected periodically (hours, weeks, and years). Bacterial survival percentages were calculated by comparing CFU from water samples to CFU from the initial LB inoculum. Blue and yellow backgrounds represent survival rates above and below 50%, respectively. **c)** Total bacterial mRNA extracted from microcosms. **d)**Quantification of bacterial transcriptional activity in freshwater, with *gapA*(glyceraldehyde-3-phosphate dehydrogenase) expression measured by RT-qPCR. **e)**Plasmid retention during freshwater incubation, assessed by PCR detection of toxin genes carried on plasmids in E2265 (LT+ and STh+) and H10407 (LT+, STh+, STp+). Bacterial colonies retrieved from microcosms after overnight growth on LB plates were subjected to PCR. “2” indicates positive PCR results for both E2265 and H10407, which encode LT and STh, while “1” indicates positive results for STp, which is unique to H10407. In all panels, data represent the median and standard error of the mean (SEM) from three independent experiments.

To assess the impact of short- and long-term freshwater exposure on ETEC, we investigated how incubation in freshwater microcosms affects bacterial survival, transcriptional activity, and plasmid retention over a period of up to 2 years. Culture-based quantification of CFU/ml (Figure 1a, b) revealed that significant decline in bacterial survival (>50%) occurred at the initial time point (0 h), when cells were first introduced to the freshwater microcosm after growth in LB-rich media followed by a relatively stable survival rate the first week.

Viability fluctuated throughout the 2-year incubation, with both strains displaying peaks at weeks 37 (39%) and 42 (53%) for H10407 and E2265, respectively (Figure 1a, b). Stadistical analysis showed no significant difference in mean CFU counts (p = 0.8331) of decline rate with 57.85% of the variance explained by strain, time, and time/strain interaction (Figure S1). After 2 years of freshwater exposure, only 0.02% and 0.005% of the bacterial cells from strains E2265 and H10407, respectively, remained culturable.

Long-term incubation markedly affected total mRNA recovery and transcriptional activity. After 70 days, both strains exhibited a 20-fold reduction in total RNA production (Figure 1d), accompanied by a 12- to 16-fold downregulation of the gene expression of glyceraldehyde 3-phosphate (G3P) dehydrogenase encoded by *gapA* compared to the LB control. By the 2-year mark, total mRNA levels increased two- to four-fold for E2265 and H10407, respectively, though *gapA* expression remained 18-fold lower (Figure 1d).

Given that ETEC toxin genes such as *eltAB, estA3/4,* and *estA1* are encoded in plasmids, we assessed the plasmid retention throughout the 2-year incubation in freshwater by PCR. We confirmed that bacterial colonies recovered from the freshwater microcosms remained PCR positive for all toxin genes at all time points, indicating plasmid stability and preservation of virulence properties, even under prolonged freshwater exposure (Figure 1e).

### Incubation in freshwater induces profound transcriptional changes in ETEC at early time points

To explore the temporal initial transcriptional response of ETEC to freshwater environment, transcriptome analyses were performed on samples collected at different time points. Cultures were harvested from the mid-log phase (3 hours) in LB broth at 37°C, and from freshwater microcosms at 23°C (± 0.5°C) at 0 h, 2 h, 24 h and 48 h post-inoculation. These time points were chosen to capture transcriptomic activity during growth in nutrient-rich LB medium at 37°C (similar to conditions in the gastrointestinal tract of mammalian hosts); the immediate transition to a nutrient-poor and ambient temperature freshwater environment (0 h), the early transcriptional response to freshwater (2 h) and adaptive responses at later stages (24 h and 48 h).

A total of 30 RNA samples (three biological replicates per time point per strain) were subjected to RNA-sequencing, generating approximately 90 million Illumina sequence reads across all samples. Pearson correlation coefficients, hierarchical clustering, and principal-component analyses (PCA) (Figure S2a and b) were used to assess transcriptome similarity between ETEC strains and across time points. The transcriptomes of LB and 0 h samples showed high intra-strain similarity, but moderate differences between the two strains. At 2 h, the transcriptomes of H10407 and E2265 appeared more correlated. The 24 h and 48 h samples exhibited the greatest transcriptional divergence from earlier time points, while maintaining high similarity to each other in both strains (Figure S2a and b).

**Figure 2:**
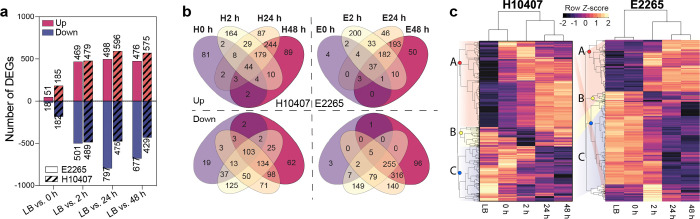
Long-term freshwater incubation extensively alters the ETEC transcriptome. **a)** Bar plot showing the number of differentially expressed genes (DEGs) in ETEC strains across the time points, with LB as the control. Magenta bars represent upregulated genes, and blue bars represent downregulated genes. **(b)** Venn diagram illustrating the number of specific and shared DEGs for each ETEC strains across the time points. Separate diagrams are shown for upregulation (“Up”) and downregulated (“Down”) genes for each strain. **c)** Heatmap displaying all DEGs identified in H10407 (1707 DEGs) and E2265 (1813 DEGs) across the time points, including the control (LB). Genes and samples were hierarchically clustered using Euclidean distance for gene expression and correlation for samples. The heatmap color corresponds to per-gene Z-score, calculated from log10-transformed read counts (after adding 0.01). Major clusters of gene expression patterns such as A (red), B (yellow) and C (blue) were colored and compared between ETEC strains.

Next, we identified the differentially expressed genes (DEGs) in each ETEC strain using DESeq2 with a cutoff of log2 fold change > |2| and an adjusted p-value (padj) of 0.01 (Table S1-S5). Transcriptomes from LB cultures served as the control. As a result, a total of 1,813/5,080 genes in E2265 and 1,707/4,972 genes in H10407 were significantly differentially expressed at any time point. As shown in Figure 2a, initial exposure to freshwater (0 h) resulted in fewer DEGs (<200 genes), while at 2 h and beyond, approximately 20% of the genome exhibited significant alterations in gene expression. Strain E2265 showed stronger transcriptional repression at 24 h and 48 h compared to H10407, although both strains upregulated a similar number of genes.

Venn diagrams were generated to compare unique and shared DEGs across time points (Figure 2b, Table S6-S7). At 2 hours, both strains showed the highest number of uniquely upregulated and downregulated genes, followed by additional unique upregulation at 48 h and downregulation at 24 h. A large overlap in up- and downregulated genes was observed between the 24 h and 48 h time points, while fewer genes were shared between 0 h and the later time points, indicating distinct temporal transcriptomic responses. A strain-specific response was evident at 0 h, where H10407 uniquely upregulated 81 DEGs, compared to just 4 in E2265.

Hierarchical clustering of DEGs (Figure 2c) found in both strains revealed time-dependent transcriptional responses. Clusters A, B, and C demonstrated progressive upregulation (Cluster A) or downregulation (Cluster C) over time, with a transient downregulation at 2 h (Cluster B), in addition, a transient upregulation at 2h clustered in cluster A in H10407 and separately in E2265 (Fig 2c). Correlation analysis showed that the transcriptomes at 0 h were most similar to the LB control, while the 24 h and 48 h time points showed closer similarity to 2 h (Figure S1a and b).

Overall, these data reveal profound transcriptomic reprogramming in ETEC strains in response to freshwater exposure, with conserved transcriptional shifts between the two strains occurring as early as 2 h post-exposure.

### Transcriptomics response to freshwater reveals ETEC’s dynamic shift between anabolic and catabolic pathways for growth and survival

**Figure 3:**
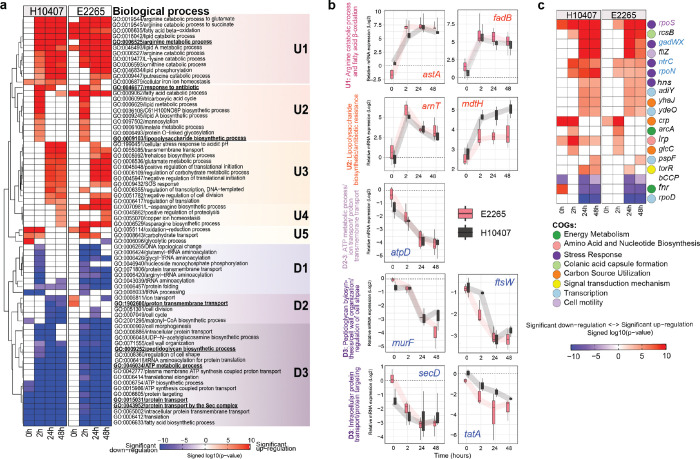
Transcriptional and functional dynamics of ETEC during freshwater incubation. **(a)** GSEA heatmap of consensus upregulated and downregulated gene sets from E2265 and H10407 using the GO term biological process as gene-sets and Piano consensus scores which were significantly enriched in both ETEC strains at each time point. Each gene set was calculated using the reporter features method including directionality (either upregulated or downregulated but not both). **(b)** Gene expression analysis by qRT-PCR for selected DEGs from RNA-seq. Box plots show relative mRNA expression values with interquartile ranges and error bars, while trend lines illustrate changes in median expression over time. **(c)** GSEA heatmap of upregulated and downregulated gene sets using RegulonDB database and Piano consensus scores which were significantly enriched in both ETEC strains at each time point. **(a)** and **(c)**, Each cell of the heatmap represents −log10 *P*-value times the sign of log-10-fold change, *i.e.,* + for upregulation (red) and – for downregulation (blue). Major clusters of gene sets are labeled with U (upregulated) or D (downregulated).

To elucidate the biological processes significantly enriched among differentially expressed genes (DEGs) and to identify their dynamic expression patterns in both ETEC strains, we conducted Gene Set Enrichment Analysis (GSEA) of Gene Ontology (GO) biological processes (Figure 3a, Tables S8–S18). GSEA identified 72 significantly enriched GO terms (FDR < 0.05), which were clustered into five upregulated and three downregulated temporal patterns (Figure 3a). As is shown in Figure 3a, the GSEA analyses revealed a higher number of upregulated biological processes compared to downregulated ones across all time points.

Subcluster U1, encompass pathways induced after 2 h of freshwater exposure and continuously upregulated over 24 and 48 hours. Up-regulated genes included amino acid degradation (e.g., *astCADBE* operon for arginine degradation), lysine degradation (*ygjG*, *prr*, *csiD*, *lhgD*), and fatty acid β-oxidation (*fadIJ*, and *fadAB*). By qRT-PCR of key genes such as *astA* and *fadB*, we confirmed the RNA-seq findings (Figure 3b), thereby validating the activation of these pathways involved in facilitating nitrogen and carbon assimilation. The activation of iron storage genes (*bfr* and *ftnA*) from the GO term cellular iron ion homeostasis exhibited strain-specific expression patterns in subcluster U1, with earlier activation in H10407 and sustained expression in E2265 (Figure 3a).

While several pathways were upregulated to obtain essential energy and biosynthetic precursors necessary for adaptation to nutrient-limited freshwater environments, other biological processes showed consistent downregulation over time, including energy metabolism, bacterial growth, transport, and membrane lipid homeostasis as shown in subcluster D3 in Figure 3a. For example, ETEC gradually downregulated the proton-translocating ATPase operon (*atpIBEFHAGDC*), which is involved in ATP metabolism, ion transport, and proton transmembrane transport, indicating a reduced need for cellular ATP synthesis during freshwater incubation. This was confirmed by a 20–30-fold downregulation of *atpD* at time 24 h and 48 h compared to growth in LB (Figure 3b). Furthermore, the two protein translocation systems, Sec (*secA*, *secYE*, *secDF*) and Tat (*tatABC*, *tatE*), responsible for transporting unfolded and folded proteins across the cytoplasmic membrane, respectively, were also downregulated, as confirmed by qRT-PCR targeting *secD* and *tatA* (Figure 3b).

Freshwater exposure resulted in the cessation of fatty acid biosynthesis by downregulating anabolic processes while simultaneously activating catabolic processes. Our data also revealed that several proteins involved in peptidoglycan biosynthesis, such as ligases (*murC, murD, murE, murF, ddlB*) and transferases (*mtgA, mraY, ftsW*), which are essential for cell wall organization and regulation of cell shape, were significantly decreased by 3–4-fold after 2 hours of incubation (Figure 3b). This downregulation indicates halted cell division and adaptive strategies to cope with the osmotic stress of the freshwater environment.

### Transient transcriptomic reprogramming at 2 hours of freshwater exposure.

At 2 hours of freshwater exposure, ETEC undergoes a transient transcriptomic response characterized by the simultaneous activation of anabolic pathways and repression of growth-related processes. Specifically, subcluster U2 exhibited significant upregulation of tricarboxylic acid (TCA) cycle genes (*aceB, aceK, fumC, glcB, gltA, mdh, sdhA–D,* and *sucA*), indicative of activation of the glyoxylate shunt to induce glyconeogenesis in response to carbon starvation (19, 20). Concurrently, ion transport genes (*actP, kdpA, kefB, mntH, putP*, and *zupT*) and lipid A biosynthesis/modification genes (*arnBCADTEF*, and *mdtH*) were upregulated (Figure 3b), supporting cellular homeostasis and alterations of the outer membrane, these processes are also enhancing antibiotic resistance mechanisms by lipid A modifications that confer resistance to cationic antimicrobial peptides (CAMPs) such as colistin and polymyxin. Quantitative RT-PCR confirmed the upregulation of *arnT* (30-fold at 2 hours and 5-fold at later time points) and *mdtH*, aligning with the transcriptomic data. In contrast, the *ampC* gene, encoding AmpC β-lactamases, was significantly downregulated in E2265 across all time points (Figure 3b).

Simultaneously, subclusters D1 and D2 revealed a pronounced downregulation of pathways associated with growth and protein synthesis (Figure 3a). In E2265, prolonged repression of genes responsible for DNA topological changes and DNA replication (*gyrA, topA, topB*) and several aminoacyl-tRNA synthetases (glutamyl, glycyl, arginyl-tRNA synthetases) was observed, suggesting increased DNA supercoiling and a reduction in replication and protein synthesis activities. This downregulation likely reflects an adaptive strategy to conserve energy and resources under nutrient-limited and stressful freshwater conditions, thereby shifting the bacterial focus from active growth to maintenance and survival.

### Late-stage transcriptomic strategy and strain-specific regulation in ETEC.

After 24 hours of freshwater exposure, ETEC exhibited a specific late-stage adaptive transcriptomic response in subcluster U3 in Figure 3a. This response involved the activation of pathways related to energy metabolism (carbohydrate metabolism), stress responses (SOS response, acidic pH adaptation, trehalose, and glutamate accumulation), and growth regulation (negative regulation of cell division and stringent translation control). Additionally, numerous genes involved in osmoregulation were upregulated, including potassium transporters (*kdpA, potH, potA*), magnesium transporters (*mntH*), amino acid transporters (*ygaV, putP*), and efflux pumps (*mdtEF*).

Strain-specific regulatory responses were also observed across subcluster U3–5 and D1–2. In the subcluster U3 from the clinical isolate E2265, the GO term “regulation of transcription, DNA-templated,” encompassing over 80 genes involved in gene-specific transcription regulation, was transiently activated at 2 hours. In contrast, the prototype strain H10407 maintained this regulatory activity from 24 to 48 hours. Furthermore, pathways related to growth and transport, such as replication and cell division (cell cycle, cell division) and ion and proton transmembrane transport, were significantly downregulated in H10407 at 24 and 48 hours. Conversely, E2265 exhibited a variable temporal pattern, including significant activation of the proton transmembrane transport system at 0 hours (D2). Moreover, subclusters U4 and U5 encompassed both shared and strain-specific pathways. Subcluster U4 involved the conserved upregulation of proteolysis regulation (*rssB*) and copper ion homeostasis (*cutC*) at 48 hours, alongside strain-specific pathways such as L-asparagine biosynthesis and carbohydrate transport. Subcluster U5 showed early and consistent activation of oxidation-reduction processes, while strain-specific pathways included glycolysis, which was significantly upregulated in H10407 at 0 hours but downregulated in E2265 at later time points, indicating divergent metabolic strategies between the two strains (Figure 3a).

### Adaptive regulatory mechanisms enhancing ETEC survival in freshwater

Most transcriptomic changes in ETEC during freshwater exposure require efficient genetic regulation to precisely activate or repress biological processes in response to environmental stimuli. By using GSEA using the E. coli K-12 Transcriptional Regulatory Network (RegulonDB) we identified 39 and 38 significantly enriched regulons (p < 0.001) in the E2265 and H10407 transcriptomes, respectively, with 19 regulons being significantly enriched in both strains (16 upregulated and 3 downregulated) (Figure 3c, Table S19–26). Our data showed that freshwater incubation induced profound alterations in the regulation of stress response, energy metabolism, and carbon utilization as a product of early activation of several stress response regulators, such as RpoS, NtrC, RpoN, GadWX, Hns, and YdeO. The concurrent activation of NtrC (nitrogen starvation response) and RpoS (nitrogen assimilation gene promoter recognition) suggests that ETEC encounters a nitrogen-limiting environment upon freshwater exposure, prompting rapid activation of NtrC-regulated genes to facilitate nitrogen scavenging from alternative sources, such as arginine catabolic pathways observed in the GSA analysis in U1 subcluster. The activation of AdiY, a positive regulator controlling the expression of arginine decarboxylases (adi) under acidic pH and anaerobic conditions, was also observed as well as the *ast*-operon induced by stationary phase (RpoS regulon) and carbon starvation (21).

Alternative sigma factors play a crucial role in transcriptional reprogramming in response to environmental conditions. The activation of growth-dependent sigma factors RpoS and RpoN, alongside the repression of RpoD, indicates that freshwater exposure alters promoter selectivity and induces transcriptional reprogramming towards stationary-phase gene expression. After 24 hours, bacterial cells exhibited increased activity of the GadWX and YdeO regulons –essential for proton-consuming acid resistance (AR) mechanisms. RpoS is also critical for the expression of GadWS and the glutamate-dependent acid resistance (GDAR) system in *E. coli* (Figure 3c).

Furthermore, several global regulons, such as ArcA, H-NS, CRP, Lrp, and Fnr, were differentially significant at various time points, supporting the notion of extensive transcriptional changes across numerous genes and metabolic pathways following two hours of freshwater incubation. For instance, major regulators of catabolite-sensitive operons displayed significant activation following the transition from nutrient-rich media to freshwater, leading to pronounced changes in carbon utilization. Similarly, ArcA and Lrp regulons were highly active at 2 hours, inducing global shifts in respiratory and fermentative states, metabolism, virulence, and motility. Conversely, the Fnr regulon was downregulated at later time points, suggesting that ETEC has not fully transitioned to anaerobic metabolism and that oxygen-free molecules may still be present in the freshwater environment (Figure 3c).

These findings highlight that ETEC strains employ distinct regulatory strategies during freshwater exposure, balancing energy metabolism, stress responses, and growth regulation to enhance survival and resilience in nutrient-limited environments. Specifically, E2265 exhibited transient transcription regulation activation at 2 hours, whereas H10407 maintained regulatory activity from 24 to 48 hours, highlighting nuanced adaptation mechanisms between strains.

### Freshwater conditions repress virulence and induce genes involved in plasmid transfer

**Figure 4:**
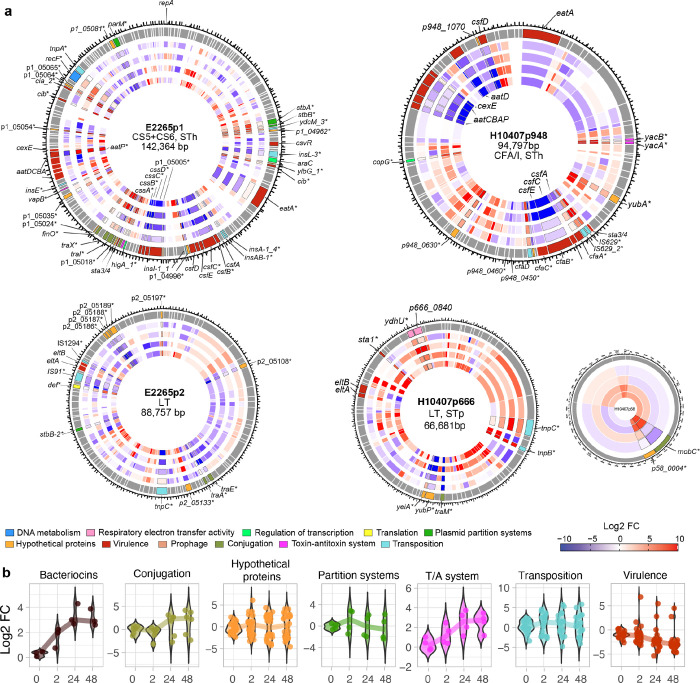
Impact of Freshwater Stress on Plasmid-Encoded Genes in E2265 and H10407. **a)** Circular heatmaps showcasing the expression patterns of plasmid-encoded genes in E2265 plasmids p1 and p2, and H10407 plasmids p948, p666, and p58. The outermost circle represents genome annotation, while the concentric circles moving inward correspond to the time points at 0-, 2-, 24-, and 48-hours post-exposure. Red tones indicate upregulation, and blue tones indicate downregulation of gene expression. Key biological processes in the outmost circles are color-coded for clarity. Differentially expressed genes (DEGs) are labeled according to their associated biological processes, with gene names marked with an asterisk (*) indicating significant differential expression. **(b)** Violin plots illustrating the gene expression levels (log₂ fold changes) of plasmid-encoded genes categorized by biological process across different time points. Trend lines depict the changes in median expression over time, highlighting the overall distribution and central tendency of gene expression levels.

To assess whether the freshwater environment enhances or represses the expression of plasmid-borne genes of ETEC, we mapped the transcript levels of plasmid-borne genes onto the plasmid sequences of E2265 (p1 and p2) and H10407 (p948, p666, and p58), as illustrated in Figure 4a and Tables S27–28. Overall, plasmid genes exhibited similar expression patterns over time, with a slight tendency toward upregulation at later time points, primarily in H10407p58 and to a lesser extent in H10407p666 (Figure S3). A total of 56 and 30 differentially expressed genes (DEGs) were identified in E2265 and H10407, respectively. Remarkably, 17 out of 19 DEGs associated with ETEC virulence were drastically downregulated in freshwater. These genes include the colonization factor operons encoding CS5 (*csfBC*) and CS6 (*cssABCD*) in E2265, and CFA/I (*cfaACE*) in H10407, along with the CFA/I regulator *cfaR*, *eatA* encoding a serine peptidase, the Aat type 1 secretion system encoded by the *aatPABCD* operon, and *cexE,* a putative virulence factor. In contrast, *aatP* in E2265p1, encoding a permease, was upregulated during freshwater exposure, reaching the highest expression level at 2 hours. Among the ETEC toxins, *sta1* encoding STp was increased 2.7-fold at both 24 and 48 hours, whereas *sta3/4* and *eltAB* showed a downward trend.

Similarly, freshwater incubation led to the repression of plasmid partition systems (Figure 4a and b). For instance, the Par and StbAB systems, including *parM* (from p1) and *stbB* (from p2), were significantly downregulated, especially at 48 hours (2-fold decrease). Conversely, *stbA* and *stbB* from E2265p1 were upregulated 2-fold at 2 hours post-incubation but exhibited opposite expression patterns at later time points, with *stbB* remaining high while *stbA* declined. No significant expression of plasmid partition systems was observed in H10407 plasmids.

In terms of activated mechanisms, genes solely present in E2265p1 involved in bacteriocin production (*cib* and *cia*) were significantly upregulated by approximately 3-fold at 24 and 48 hours (Figure 4a and b). Similarly, plasmid-encoded toxin-antitoxin (T/A) systems in E2265p1 and H10407p948, such as the RelE-RelB system ribosome-dependent mRNA interferases (*yacB* and *yacA*) and the VapB antitoxin from the VapC-VapB system, as well as gp49 encoding a prophage, were upregulated by 2.6 to 2.7-fold at 24 and 48 hours. The results suggest that ETEC initially represses bacterial conjugation at 2 hours but later promotes plasmid transfer through active pilus formation (*traX*) and enhanced plasmid transfer (*traI*), along with modulation of horizontal gene transfer via upregulation of the regulatory protein *finO* in E2265p1. In H10407p58, *mobC* was upregulated at 24 and 48 hours, indicating activation of plasmid mobilization. In contrast, pilus-related genes of the Type IV conjugative transfer system (*traE* and *traA*) in E2265p2 were repressed.

Transposase gene expression generally increased at 2 hours but remained low thereafter, with significant variability across plasmids. In E2265p1 and H10407p948, some transposases were activated after 2 hours of incubation. Additionally, a large proportion of hypothetical genes exhibited varied levels of differential expression with unknown functions, warranting further investigation.

Therefore, this data suggests that bacteria repress virulence and plasmid maintenance under freshwater stress while enhancing plasmid transfer mechanisms competitive fitness and stress resistance that might facilitate bacterial survival and persistence in nutrient-limited and stressful environments.

### Freshwater incubation primes ETEC for biofilm formation in a strain-specific fashion but cause substantial morphologic changes.

Pathogens are typically introduced into water systems through human fecal contamination, where they establish themselves within self-produced extracellular matrices and attach to biotic or abiotic surfaces, forming biofilms (23). Our GSA of transcriptomic data revealed a significant upregulation of genes involved in single-species biofilm formation (GO:0044010) in the H10407 strain, while the E2265 strain showed a less pronounced response (Table S16) over time. To investigate whether this transcriptional trend translates to phenotypic changes, we assessed biofilm formation following extensive freshwater incubation, considering that ETEC can survive for months to years in water.

**Figure 5:**
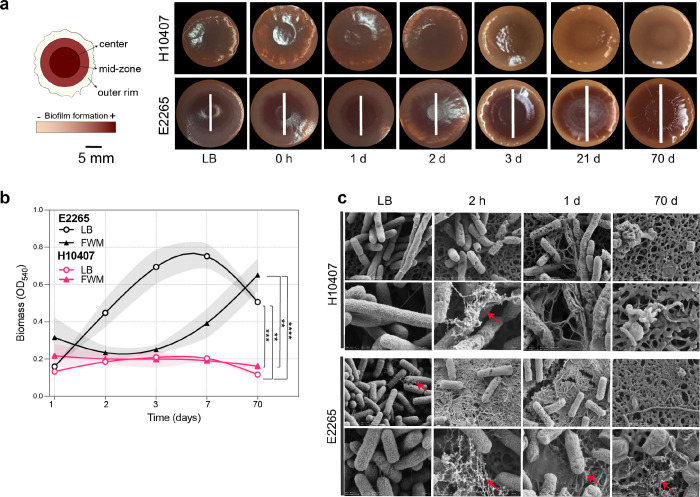
ETEC biofilm formation and cell wall integrity under freshwater exposure. **a)** Macroscopy images of colony biofilm phenotypes of H10407 and E2265 on Congo Red (CR) agar medium without salts after 48h of growth at 23°C. Freshwater microcosm samples were collected at different time points and inoculated on CR-assay plates. Scale bar represents 5 mm. Schematic overview of the macrocolony and zone differentiation. The intensity of the CR stanning correlates with biofilm component production (Illustration modified from (22)): brown pigmentation indicates curli expression. **b)** Quantification of biomass accumulation using Crystal violet (CV) stanning overtime in LB culture and freshwater microcosms. Lines represent the mean, dots indicate measurements for four biological replicates, and error bars denote the standard error of the mean. P-values were derived from two-way ANOVA with Turkey’s multiple comparison correction (*, *P* <0.5; **, *P* < 0.01; ***, *P* < 0.001; ****, *P* < 0.0001). **c)** Representative Scanning Electron Microscopy (SEM) images of H10407 and E2265 strains incubated in freshwater microcosms at different time points. Red arrows show a dense structure around cell bodies originating from curli/fimbriae and EPS production. Magnifications and bar markers are x40,000 (upper panel) and x80,000 (lower panel). Scale bars of the inset micrographs = 200nm.

Biofilm formation was quantified using Congo Red (CR) assays, Crystal Violet (CV) assays, and Scanning Electron Microscopy (SEM) on samples collected from freshwater microcosms at various time points up to 70 days. As depicted in Figure 5a, the CR-assay demonstrated distinct colony phenotypes between H10407 and E2265 when grown on CR agar medium without salts after 48 hours of growth at 23°C. H10407 exhibited smooth, white (saw) colonies with no CR pigmentation, whereas E2265 formed brown, dry, and rough (bdar) macrocolonies with strong red pigmentation in the center, a less pigmented mid-zone, and an extensive white outer rim. The bdar morphotype indicates increased production of curli, which mediates biofilm formation. Over time, E2265 macrocolonies displayed a trend of enhanced biofilm formation, characterized by higher CR pigmentation in the center and mid-zone and a significant reduction of the outer rim. This trend was most pronounced in macrocolonies from the 70-day freshwater incubation period, which exhibited a wrinkled phenotype associated with high cellulose expression. In contrast, H10407 macrocolonies did not show any significant increase in biofilm formation (Figure 5a).

These observations were corroborated by the CV-assay results (Figure 5b), which showed a two-fold increase in biofilm biomass accumulation in E2265 after 70 days incubation in freshwater microcosms. In LB media, E2265 exhibited significantly higher biofilm production after 3 and 7 days of incubation (P < 0.05) compared to freshwater conditions, but drastically reduced at 70 days. H10407 consistently showed significantly lower biomass production in both control and freshwater microcosms (P < 0.001).

To further characterize biofilm formation, SEM was performed on ETEC samples grown in LB and freshwater microcosms at 2 hours, 1 day, and 70 days (Figure 5c). SEM images revealed a greater presence of extracellular polymeric substances (EPS) and fibers resembling curli in E2265 at 2 hours and 24 hours of freshwater incubation. Additionally, prolonged freshwater exposure resulted in significant alterations to cell morphology and surface integrity. Cells incubated for longer periods exhibited multiple dimples and holes on the cell wall surface as early as 2 hours, suggesting rapid osmotic shock. After 27 weeks, SEM images showed a majority of burst and lysed cells, accompanied by cell debris, indicating osmotic lysis. Abnormal cell shapes were predominantly observed in E2265 samples, including larger spherical cells with poor turgor, rod-shaped cells with slightly wider centers, and elongated cells (>3 μm) (Figure 5c).

These results demonstrate that freshwater conditions prime surviving ETEC cells, particularly E2265, to form robust biofilms through enhanced curli and cellulose production. However, prolonged exposure to freshwater also adversely affects bacterial morphology, division, and survival, highlighting a trade-off between biofilm formation and cellular integrity under nutrient-limited and stressful environmental conditions.

### Enhanced adherence of ETEC to human epithelial cells following long-term freshwater exposure

**Figure 6:**
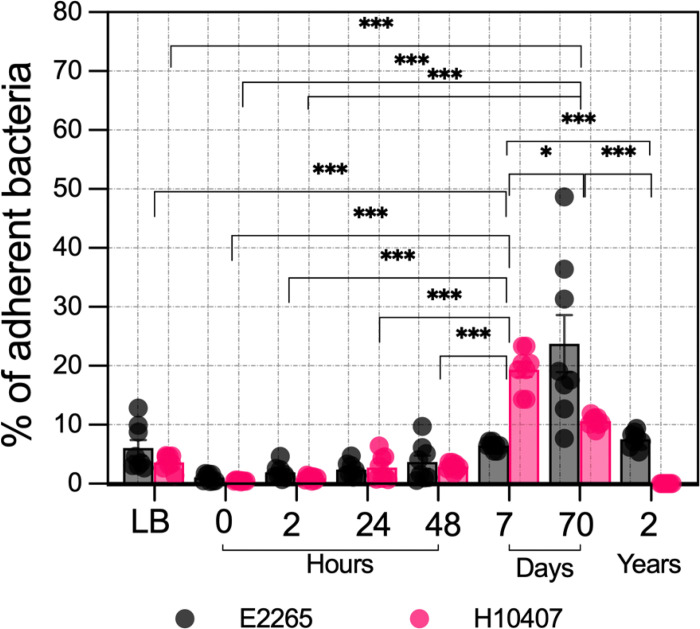
Adhesion Potential of ETEC Incubated in Freshwater. Percentage of adherent bacteria from co-culture experiments using ETEC samples collected at different time points from freshwater microcosm incubations and Caco-2/HT-29 human epithelial cell monolayers. Bars represent the mean values, dots indicate individual measurements from four biological replicates, and error bars denote the standard error of the mean (SEM). P-values were derived from a two-way ANOVA to assess the effects of strain and time on bacterial attachment, followed by Tukey’s HSD post-hoc tests to determine pairwise differences between time points (*, P < 0.05; **, P < 0.01; ***, P < 0.001).

Polluted drinking water serves as a vehicle for enteric pathogens to enter the host and colonize the gastrointestinal tract, initiating infection (24). Adherence to human epithelial cells is a crucial hallmark of ETEC pathogenesis and is highly regulated by environmental factors. Therefore, we investigated whether ETEC cells retain the ability to attach to human intestinal epithelial cells after short- and long-term exposure to freshwater. Caco-2 and HT29 human epithelial cells were challenged with ETEC samples incubated in freshwater for various time points, and the number of attached bacteria was quantified five hours post-infection (hpi). As shown in Figure 6, early incubation periods (0h to 48h) exhibited very low adherence capacity (less than 5%) compared to the control in LB media. In contrast, adherence significantly increased over time, particularly between prolonged incubation periods of 7 days and 70 weeks. Two-way ANOVA revealed a significant effect of both strain (p-adj = 0.0279) and time (p < 2e-16) on bacterial attachment to human cells (Table S29). In addition, Tukey’s post-hoc test showed significant differences between various time points.

Differences in binding ability between the two ETEC isolates were most notable after the 7-day time point. Interestingly, E2265 cells incubated for 2 years in freshwater attached to human epithelial cells, whereas H10407 samples from the same time point showed no colony-forming units after the co-culture assay. Overall, ETEC cells that exhibit greater persistence in freshwater microcosms also display enhanced adherence to human epithelial cells. This increased binding ability was more pronounced in the E2265 strain, which expresses CS5 and CS6 colonization factors.

### Freshwater exposure select for colistin-resistant subpopulations in ETEC

To validate transcriptomic findings that freshwater exposure induces the overexpression of the *arn* operon—linked to lipid A modification and enhanced resistance to colistin—we conducted Population Analysis Profile (PAP) assays across a gradient of colistin concentrations. Initial MIC assessments using the broth microdilution method in Mueller-Hinton (MH) broth revealed that both ETEC strains, E2265 and H10407, exhibited a MIC of 1 mg/L, classifying them as colistin-sensitive since this value is below the clinical breakpoint of 2 mg/L. Importantly, no “skip wells” were observed, indicating the absence of resistant subpopulations within the same culture under standard conditions.

**Figure 7:**
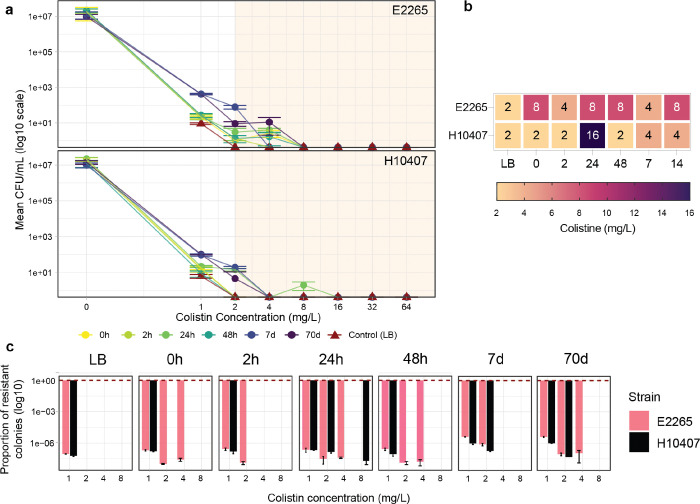
Impact of freshwater exposure on colistin resistance in ETEC strains E2265 and H10407. **(a)**Population Analysis Profile (PAP) of E2265 and H10407 strains incubated in freshwater microcosms at various time points (0, 2, 24, 48 hours; 7 and 70 days). Colistin concentrations (mg/L) are indicated on the x-axis. The number of colistin-resistant colonies (CFU/mL) is plotted, with orange shading highlighting concentrations above the clinical breakpoint for ETEC strains. **(b)**Heatmap illustrating the variation in Minimum Inhibitory Concentration (MIC) across different time points for E2265 and H10407 strains. **(c)** Bar plot of the proportion of colistin-resistant subpopulations in E2265 and H10407 strains at each time point. All PAP assays were performed in triplicate.

Subsequently, samples from control (LB) cultures and freshwater microcosms at various time points (0h, 2h, 24h, 48h, 7 days, and 70 days) were subjected to PAP assays to quantify the proportion of colistin-resistant colonies. This involved serial dilution and plating on LB agar without antibiotics to determine total bacterial populations (CFU/mL), alongside plating on LB agar supplemented with colistin at concentrations of 1, 2, 4, 8, 16, 32, and 64 mg/L. Under control conditions, the PAP assays revealed an exceedingly low proportion of colistin-resistant colonies, ranging from 5.9 × 10^−8^ to 8.6 × 10^−8^ at the MIC of 1 mg/L, with no resistant colonies detected at higher antibiotic concentrations (Figure 7a–c).

In contrast, bacteria exposed to freshwater environments exhibited subpopulations of colistin-resistant colonies capable of surviving antibiotic concentrations up to 8 mg/L (4-fold above the clinical breakpoint) (Figure 7a and 7b, S Figure 4 and 5, S Table 30). Although the proportion of these resistant colonies remained below 10^−6^ relative to the initial bacterial load in LB, a notable increase was observed in E2265 strains at higher colistin concentrations. Specifically, at 24h and 48h—time points corresponding to active expression of the *arn* operon—resistant colonies were detected at 2-fold and 4-fold higher MICs, respectively. Also, E2265 incubated for 7 and 70 days displayed higher bacterial counts and proportions of colistin-resistant colonies (Figure 7a and c). The highest concentration at which a subpopulation of H10407 survived was 8 mg/L at the 24-hour freshwater microcosm; however, this resistance pattern was unstable, as samples from later time points did not sustain growth at the same concentrations. Despite regression analysis indicating no significant differences between strains H10407 and E2265, visual inspection of the PAP data suggested that E2265 may harbor a slightly higher number of resistant colonies at elevated colistin concentrations. This trend hints at potential strain-specific resistance mechanisms, although it was not statistically validated in the current model. These findings demonstrate that while freshwater environments drastically reduce overall bacterial viability, a minor subset of bacterial cells develops enhanced resistance to colistin. This resistance varies between ETEC strains, with E2265 showing a higher number of resistant colonies at increased antibiotic concentrations. Prolonged incubation in freshwater environments may further facilitate bacterial tolerance to antibiotics, potentially through mechanisms such as lipid A modification.

## DISCUSSION

In this study, we examined the transcriptomic responses and survival strategies of ETEC strains E2265 and H10407 during short- and long-term freshwater exposure, providing insights into their adaptive mechanisms during transition from the human host (37°C and high levels of nutrients) to the environment, such as freshwater (22°C and low level of nutrients). To understand how enterobacterial pathogenic species mitigate hypo-osmotic stress we abruptly shifted ETEC grown at mid exponential phase in LB to sterile-filtrated fresh water. Freshwater shock immediately resulted in 50% reduction of CFU but after the initial reduction ETEC cells remained viable for prolonged periods. Several studies have shown that *E. coli* and related enterobacterial species can survive and even grow in nutrient limited water environments (9, 25–30).

The initial response followed by adaptation to nutrient limiting and osmotic and temperature challenging conditions has been shown to be accompanied by large changes in the transcriptome. A large part of this survival response is directed by the global stress response regulator rpoS (28, 30). Similar to our results where a third of the ETEC genome was altered, a transcriptome study in *Salmonella enterica* serovar Typhi determined transcription of several timepoints during the first 24 hours of water incubation and found differential expression in over half of the genome (29). Entry into water induced genes involved in the glycolysis and the TCA cycle in accordance with the results after 2 hours in the present study. These changes together with activation of arginine and lysine degradation and downregulation of anabolic pathways reflects the shift to halted growth and cell division and adaptations to secure carbon and energy. For instance, arginine, a key energy- and nitrogen-rich amino acid, can serve as a sole source of nitrogen through degradation by the succinyltransferase pathway (Ast) under nitrogen-limiting and aerobic growth conditions (31). The activation of fatty acid β-oxidation pathways (*fadIJ* and *fadAB*) highlights a metabolic adaptation aimed at generating energy and acetyl-CoA from fatty acid breakdown (32). The production of acetyl-CoA through fatty acid β-oxidation feeds directly into the tricarboxylic acid (TCA) cycle for ATP production (33).

We found involvements of several global and major transcription factors including ArcA, H-NS, CRP, Lrp, and Fnr. An upregulation of the ArcA and Lrp regulons was recorded at 2 hours. The ArcAB regulon is linked to starvation and stress responses and well as compromised membrane integrity responses mediated by the phage shock protein (Psp) system. In line with this an > 16-fold upregulation of the *pspABC* operon was observed in the two isolates analyzed in this study. Phage shock genes were also among the most highly upregulated genes at 24 h after entry into water in the Kingsley’s study (29). These genes encode proteins essential for survival under nutrient and energy-limited conditions by maintaining proton motive force and intact inner membranes.

The activation of RpoS, YdeO, and GadWX regulons during the first 24 hours of water adaptation is mainly directed towards coping with envelope stress. These operons are also key factors in coping with acid stress (34–36). The highest induced gene found in the entire present dataset was *yciG* with a more than 1,000-fold upregulation after 24 and 48 h in water compared to LB in both H10407 and E2265. YciG is involved in biofilm formation and positively regulated by McbR (37). YciG was found to be involved in acid stress in *Salmonella enterica* Typhimurium where the *yciEFG* operon was found to be 1.000 fold upregulated during acid stress while *yciG*-mutants showed inability to survive acidic pH and with reduced adherence (35). These results indicate that the acid stress response and water stress responses are similar and mainly directed towards envelope preservation and linked to biofilm formation.

Hypo-osmotic stress results in an influx of water molecules which increases cellular turgor pressure. The ruptured membranes seen after prolonged water exposure reflects inability to mitigate the turgor pressure. This study also showed that ETEC cells underwent morphologic changes which were examined using scanned electron microscopy. The SEM images confirmed cell disruption, and exopolysaccharide matrix, a common feature during biofilm formation. Changes in the composition and structure of the membrane are responses to the osmotic pressure (38). Sudden changes in the osmotic pressure leads to disruption of the cell wall integrity and loss of the rod-shaped morphology which result in bacterial burst and dead (39). These results are in accordance with our observation that the viability of cells decreased rapidly during the initial response and at a slower pace during the long-term experiment when bacterial cells adapted. In accordance with adaptation over long periods we observed that a subset of surviving cells after long-term incubation in water gradually adapted towards better adhesion to epithelial cells and biofilm formation capacity as well as acquisition of colistin tolerance. The Population Analysis Profile (PAP) assays revealed a significant increase in colistin-resistant subpopulations in freshwater-exposed ETEC samples, particularly in strain E2265. Polymyxins, including colistin and polymyxin B, are lipopeptide antibiotics commonly used to treat multidrug-resistant Gram-negative bacterial infections. The overexpression of these genes was shown to confer resistance to several cationic antimicrobial peptides (CAMPs) in gram-negative bacteria, including colistin and polymyxin (40, 41). A well-documented mechanism of polymyxin resistance in *Escherichia coli* and other closely related Enterobacteriaceae involves the modification of lipid A, a component of lipopolysaccharide (LPS), by the addition of positively charged residues such as 4-deoxy-L-aminoarabinose (Ara4N, encoded by the *arn* operon) and phosphoethanolamine (PEtN, encoded by *ept/pmr* genes) (42). These modifications reduce the binding efficacy of polymyxins to LPS by neutralizing its negative charge. The expression of these modifications is controlled by a complex regulatory network that responds to environmental stimuli such as metal ion concentrations, pH, cationic peptides, and outer membrane fluidity (43). Increased *arn* operon expression has been observed in colistin-resistant bacteria such as *Proteus* and *Serratia* species (40). In addition, the acquisition of an IncFII plasmid encoding an extra copy of the *arn* operon has been shown to confer heteroresistance to colistin in previously susceptible *E. coli* strains (44).

In line with our findings of evolving colistin tolerance after water adaptation a study on enterohemorrhagic *E. coli* (EHEC) adapted to acidic conditions developed polymyxin and colistin resistance (45). Comparison of the transcriptome of acid-adapted EHEC isolates to non-adapted isolates revealed upregulation of stress resistance genes *kdpA* and *bshA*, as well as *arnA* (45) which is in accordance with the transcriptional up-regulation of these genes during water adaptation observed in this study. The same genes were also found to be upregulated during acid stress response (46). Serial passage growth in sub-MIC colistin to produce colistin resistant subpopulations induced high expression of *arnABCDEF* and *pagE* in the colistin-adapted isolates, as well as up-regulation of magnesium transporters (MgtA and MgtE), and multidrug export protein (MdtEF) and envelope proteins YfaZ and VirK while L-arginine biosynthesis pathway and *potE* and *mgrB* were downregulated (47). Inactivation of MgrB, a negative regulator of the phoPQ signaling system induced colistin resistance in Enterobacter (47, 48).

These resistance or tolerance mechanisms are independent of the well-known *mcr-1* gene and are considered intrinsic adaptations. Our findings suggest that freshwater environments may act as selective pressure that enhances antibiotic resistance. Transcriptomic analysis revealed overexpression of the *arn* operon in ETEC strains exposed to freshwater, likely driving the observed colistin resistance through lipid A modification. Notably, the stability of colistin in water is influenced by pH, with degradation rates of less than 3% over 24 hours at pH 5 (49). Although environmental colistin concentrations are extremely low (42), this genetic adaptation in ETEC highlights the potential for freshwater environments to contribute to the emergence and spread of antibiotic-resistant pathogens.

Our results of gene expression during adaptation to water conditions share characteristics in gene expression patterns with isolates gradually adapted to both colistin/polymyxin and acid stress tolerance. These results indicate that adaptation to both water during water-borne transmission, and passage through the acidic stomach upon ingestion will make pathogenic bacteria more tolerant to membrane disrupting molecules including antibiotic treatment and antimicrobial peptides which might partly explain why diarrheal diseases often peaks during flooding and consumption of unsafe water. Virulence was however not induced in water, instead transitioning to freshwater induced a downregulation of ETEC virulence genes, likely representing a strategic reallocation of energy from virulence to survival processes under environmental stress. These results align with previous studies that indicate reduced virulence factor expression in nutrient-deprived environments (8).

Long-term incubation revealed that plasmids with toxin genes are detectable up to 2 years of incubation. We have previously shown that ETEC lineages have a remarkable stable plasmidome and the same plasmid content is found in genetically related isolates all over the world and over time (50, 51). This indicates that there is an evolutionary pressure to keep plasmids even during prolonged water stress. Toxin-antitoxin genes are key regulators of plasmid retention (52) and these systems were upregulated in both isolates in this study indicating that water stress favors plasmid stability. This might also explain why multi-drug resistant and/or pathogenic *E. coli* isolates keep their plasmids even though often recovered from wastewater and polluted environmental water sources.

One limitation of this study is the use of laboratory-controlled freshwater microcosms, which may not fully replicate the complexity of natural water bodies. Additionally, only two ETEC strains were examined, limiting the ability to generalize findings across diverse ETEC populations. Future research should explore the adaptive responses of a broader range of ETEC strains in diverse environmental conditions to validate the observed trends. Additionally, investigating the interplay between ETEC and other microbial communities in freshwater could provide deeper insights into the mechanisms driving pathogen transmission, antibiotic resistance and biofilm formation.

In conclusion, this study elucidates the complex adaptive strategies employed by ETEC in freshwater environments, highlighting critical genetic and phenotypic changes that enhance survival and antibiotic resistance. Understanding how pathogenic species like ETEC adapts to freshwater environments is essential for preventing and controlling diarrheal outbreaks. Freshwater not only serves as a reservoir but also as a transmission route for ETEC, enabling these pathogens to persist and evolve under nutrient-poor conditions. These insights are pivotal for informing public health strategies aimed at mitigating the impact of waterborne infections

## MATERIAL AND METHODS

### Bacterial strains

The bacterial strains used in this study included the clinical ETEC isolate E2265, which is CS5+CS6 LT+STh positive and carries two plasmids (p1 and p2). This strain was isolated from a Bangladeshi adult with diarrhea in 2006 (53). Additionally, the prototype ETEC isolate CFA/I LT+STh/STp positive strain H10407, isolated from an adult with cholera-like symptoms during an epidemiological study in Dhaka, Bangladesh (54), was used. H10407 carries four plasmids, designated pETEC948, pETEC666, pETEC58, and pETEC52 (55).

### Freshwater microcosm setup

#### Freshwater collection.

Freshwater samples were collected from Lake Lötsjön, Stockholm, Sweden, in March 2019. The water was filtered using a 0.4 μm syringe filter (Thermo Fisher Scientific^®^), and the pH of the filtered freshwater was measured, yielding a value of 6.5. To ensure sterility, 100 μL of the filtered freshwater was plated onto Luria Bertani agar (Thermo Fisher Scientific) and incubated overnight at 37°C. Aliquots of 50 mL sterile freshwater were stored at −20°C until use.

#### Bacterial culturing and exposure to freshwater.

The ETEC strains E2265 and H10407 were first streaked onto Blood agar plates and incubated overnight at 37°C. Ten to twelve colonies of each strain were then collected and suspended in 600 μL LB broth (Thermo Fisher Scientific). A 100 μL aliquot of this suspension was inoculated into an Erlenmeyer flask containing 50 mL of LB broth and incubated at 37°C with shaking at 150 rpm until the bacterial culture reached an optical density (OD600) of 0.3, as described in Lothigius et al. (2008). The bacterial culture was then centrifuged for 2 minutes at 4500 rpm, and the pellet was resuspended in 50 mL of filtered lake water. This suspension was transferred into a 200 mL screw-cap borosilicate glass bottle, which was sealed to prevent evaporative loss and airborne contamination. The microcosms were aerated weekly under a laminar airflow cabinet during sample collection. The bottles were stored in a temperature-controlled room (23°C) for the duration of the experiment, and samples were collected for further analyses.

#### Bacterial quantification and viability.

To quantify the viable cells of E2265 and H10407 in freshwater experiments, 20 μL samples were collected at each time point, followed by a ten-fold serial dilution in 1X PBS. From the appropriate dilution, 10 μL was plated onto LB agar plates and incubated at 37°C overnight. Bacterial viability was calculated as a percentage of the initial inoculum using the following formula: Viability (%)=[Initial CFU (inoculum)/ CFU at time point]×100. All experiments were performed in triplicate, and the results were expressed as the mean ± standard error of the mean (SEM).

#### Plasmid stability assay.

Bacterial colonies from the LB agar plates used for viability testing were selected for plasmid stability analysis. DNA was extracted using the boiling method. Specifically, five colonies from each time point were suspended in 100 μL of distilled water and boiled at 100°C for 10 minutes. The suspensions were then centrifuged at 10,000 rpm for 5 minutes, after which the pellet was discarded, and the supernatant was used as the DNA template for PCR, without quantifying the DNA concentration. PCR was performed following the protocol described by Sjöling, Wiklund (56), using primers targeting the ETEC toxins LT (*eltAB*), STh (*sta2*), and STp (*sta1*) (Table S31). PCR products were separated by agarose gel electrophoresis and visualized under a UV transilluminator.

#### RNA isolation and Quantitative Reverse Transcriptase PCR (q-RT-PCR).

During the sampling period, 600 μL of bacterial culture in LB broth (control) and from freshwater microcosms were collected. To stabilize the RNA, 1200 μL of RNAprotect Bacteria solution (Qiagen) was added to each sample. The samples were centrifuged at 10,000 rpm for 10 minutes, the supernatant was discarded, and the pellets were stored at −80°C until RNA extraction. RNA was extracted using the RNeasy Plus Mini Kit (Qiagen), following the manufacturer’s instructions. The concentration and purity of the RNA were measured using a Nanodrop spectrophotometer (Thermo Fisher Scientific^®^) and a Qubit 2.0 Fluorometer (Life Technologies) with the Qubit RNA HS Assay Kit (Invitrogen).

For RNA sequencing, total RNA was collected from bacteria grown in LB (control) and from samples at 0 and 2 hours (short-term freshwater incubation) and at 24 and 48 hours (long-term freshwater incubation). The RNA was precipitated, washed, and shipped in 99.5% ethanol to the sequencing facility at Beijing Genome Institute (BGI), Shenzhen, China. Upon arrival, RNA integrity, quality, and concentration were rechecked using an Agilent Bioanalyzer, and all samples had RIN values greater than 9.

Two-step quantitative reverse transcriptase PCR (qRT-PCR) was conducted to assess gene expression. Complementary DNA (cDNA) was synthesized from extracted total RNA using the QuantiTect Reverse Transcription Kit (Qiagen), with each sample normalized to 100 ng/μL. qRT-PCR was performed on a LightCycler 480 II (Roche) using custom-designed primers and previously published primers (Table S31). Each 25 μL reaction mixture contained 12.5 μL of Power SYBR Green PCR Master Mix (Applied Biosystems), 600 ng of cDNA, and 1 μL of each primer (forward and reverse). All assays were run in duplicate, including treatment and control groups, as well as negative controls without a template. Gene expression was normalized to the housekeeping gene *gapA* using the 2−ΔΔCt method. Cycle threshold (Ct) values were used to calculate relative gene expression, with LB media serving as the control. Results are presented as the mean of at least three independent experiments.

#### Illumina RNA-Sequencing and analysis.

RNA libraries were prepared according to the Illumina TruSeq protocol and sequenced on an Illumina HiSeq 2000 platform using 100 bp paired-end reads, following the protocols of the Beijing Genome Institute. Quality control of the paired-end reads was performed using FastQC. RNA-Seq reads from each biological sample were aligned to their respective genome sequences using Bowtie (57), with high-quality reads mapped to the reference genomes of ETEC strain E2265 (chromosome: CP023346, plasmid p1: CP023347, plasmid p2: CP023348) and H10407 (chromosome: FN649414, pETEC948: FN649418, pETEC666: FN649417, pETEC58: FN649416, pETEC52: FN649415) using HTSeq (58). Differential expression analysis was conducted using the DESeq workflow (59). To identify differentially expressed genes, we performed four comparisons: T0h vs. TLB, T2h vs. TLB, T24h vs. TLB, and T48h vs. TLB, using a log fold change (logFC) threshold of 4 and adjusted p-value (padj) cutoff of 0.01.

All raw data generated by the RNA-Seq analysis have been deposited in the Short Read Archive (SRA) under the BioProject PRJNA1221672.

#### Gene set enrichment analysis (GSEA).

Gene set enrichment analysis (GSEA) was performed using the R package Piano on log2 fold changes and adjusted p-values obtained from DESeq2, using gene-level statistics. The analysis was conducted with the geneSetStat = “reporter” setting and 1000 permutations (nPerm = 1000). Gene Ontology (GO) biological processes were sourced from MSigDB(60). GO processes were considered significantly enriched if they had a false discovery rate (FDR) below 5% and exhibited a clear directional change (i.e., not mixed). Merged heatmaps were generated using the R packages ComplexHeatmaps.

#### Minimum Inhibitory Concentration (MIC).

The MIC was conducted using broth microdilution method, following the guidelines outlined in the European Committee on Antimicrobial susceptibility testing (EUCAST). *E. coli* ATCC 25922 was used for routine quality control.

### Biofilm formation assays:

#### Crystal Violet Assay.

Biofilm quantification was performed using the Crystal Violet assay as described by Joffré et al. (2019). Overnight cultures of ETEC strains grown in LB at 37 °C with shaking were diluted 1:100 in fresh LB medium. Subsequently, 150 μL of the diluted culture was aliquoted into round-bottom 96-well polystyrene plates (Sarstedt) and incubated at room temperature (23 °C) in a humid chamber for 1, 2, 3, 7, and 70 days. Fresh media was replenished every other day as needed. For freshwater biofilm assessment, 150 μL samples from freshwater microcosms at time 0 h were similarly aliquoted into 96-well plates and sealed with parafilm to prevent evaporation. After incubation, wells were washed twice with 1X PBS to remove non-adherent cells, fixed with 75% ethanol, and stained with 0.5% crystal violet (Sigma-Aldrich). Following three washes with distilled water and air-drying, the bound dye was solubilized in 95% ethanol. Biofilm intensity was quantified by measuring absorbance at 540 nm. All assays were conducted in sextuplicate across three independent experiments, with LB-only and freshwater-only wells serving as negative controls.

#### Congo Red Test.

To assess biofilm formation, the Congo Red assay was conducted. Samples of 500 μL were collected from LB medium and freshwater microcosms at time points of 0, 1, 2, 24, 48 hours, and on the 7th and 70th days. Each sample was centrifuged at 10,000 rpm for 10 minutes, washed once with 100 μL of PBS, and resuspended. Subsequently, 1 μL of each resuspended sample was spotted onto LB agar plates without salt supplemented with Congo Red dye. Plates were sealed with parafilm and incubated at room temperature for 48 hours. After incubation, biofilm formation was visually documented by photographing the plates.

#### Scanned Electron Microscopy (SEM).

Samples were collected from LB sample and when bacterial cell grown in freshwater at different time-points to perform SEM at Karolinska Institutet, Stockholm. A 10 ml sample was collected during sampling time LB sample (control sample) and 2hour, 24hour and 27th week of bacterial growth in freshwater, centrifuged at 4500 rpm for 2 mins, washed once with 5 ml of PBS and fixed with 1500 μl fixative solution (2% paraformaldehyde + 2.5% glutaraldehyde in 0.1M Cacodylate buffer) and stored at 4°C until delivered to Karolinska Institutet for SEM analysis. SEM images were analyzed using image processing program ImageJ.

#### Population Analysis Profiling (PAP).

PAP assays were conducted to evaluate the heteroresistance of ETEC strains. ETEC strains were cultured in LB medium (as control) and in freshwater microcosms at various time points. Bacterial samples from both LB cultures and freshwater microcosms were collected, serially diluted, and plated on LB agar plates without antibiotics to quantify the total bacterial population (CFU/mL). In parallel, 100 μL from the LB bacterial culture and 1 mL from the freshwater microcosms were plated onto LB agar plates supplemented with colistin salts (Sigma-Aldrich) at concentrations of 1, 2, 4, 8, 16, 32, and 64 mg/L. These plates were incubated at 37 °C for 24 hours. The fraction of resistant mutants was calculated by dividing the number of colonies growing in the presence of colistin by the total number of cells plated on the non-antibiotic LB plates.

### Statistics:

The **multiple linear regression model** was performed using the combined data of CFU counts for ETEC strains E2265 and H10407 into a single dataset, with time points and strain labels for each observation. The effects of time and strain on CFU counts, including an interaction term to assess differences in CFU decline between strains. The model was implemented using the lm()` function in R, and results, including regression coefficients and statistical significance. The paired t-test performed to compare CFU counts between E2265 and H10407 strains at corresponding time points was conducted using the t.test() function in R, with the paired = TRUE option to account for the matched measurements across strains. All analyses were conducted in R version 2024.04.2+764 .

## Importance

Environmental conditions play a vital role in shaping the behavior of pathogenic bacteria, influencing their survival, virulence, and resistance to treatments. This study reveals how freshwater environments act as crucial reservoirs for enterotoxigenic *Escherichia coli* (ETEC), one of the most common causes of diarrhea in children, by driving genetic adaptations that enhance biofilm formation and antibiotic resistance. These adaptive changes increase resilience and ability to cause disease, posing significant public health risks by facilitating persistent waterborne infections. Understanding the environmental factors that influence pathogenic bacterial behavior is essential for developing effective strategies to prevent waterborne outbreaks and manage antibiotic-resistant infections, ultimately protecting vulnerable populations from severe diarrheal diseases.

## References

[1] Anderson JDIV, Bagamian KH, Muhib F, Amaya MP, Laytner LA, Wierzba T, Burden of enterotoxigenic Escherichia coli and shigella non-fatal diarrhoeal infections in 79 low-income and lower middle-income countries: a modelling analysis. The Lancet Global Health. 2019;7(3):e321–e30. 30784633 10.1016/S2214-109X(18)30483-2 PMC6379821

[2] Daniels NA, Neimann J, Karpati A, Parashar UD, Greene KD, Wells JG, Traveler’s Diarrhea at Sea: Three Outbreaks of Waterborne Enterotoxigenic Escherichia coli on Cruise Ships. The Journal of Infectious Diseases. 2000;181(4):1491–5. 10762583 10.1086/315397

[3] UN-Water. Summary Progress Update 2021 – SDG 6 – water and sanitation for all. Version:2021 Geneva, Switzerland.

[4] WHO. World Health Organization. Drinking Water Geneva, Switzerland World Health Organization; 2023 [updated September 19, 2024. Available from: https://www.who.int/news-room/fact-sheets/detail/drinking-water.

[5] Toledo CC, Mentzer Av, Agramont J, Thorell K, Zhou Y, Szabó M, Circulation of enterotoxigenic Escherichia coli (ETEC) isolates expressing CS23 from the environment to clinical settings. mSystems. 2023;8(5):e00141–23. 37681982 10.1128/msystems.00141-23 PMC10654058

[6] Guzman-Otazo J, Gonzales-Siles L, Poma V, Bengtsson-Palme J, Thorell K, Flach CF, Diarrheal bacterial pathogens and multi-resistant enterobacteria in the Choqueyapu River in La Paz, Bolivia. PLoS One. 2019;14(1):e0210735. 30640938 10.1371/journal.pone.0210735 PMC6331111

[7] Mero S, Lääveri T, Ursing J, Rombo L, Kofoed PE, Kantele A. Seasonal variation of diarrhoeal pathogens among Guinea-Bissauan children under five years of age. PLoS Negl Trop Dis. 2023;17(3):e0011179. 36913409 10.1371/journal.pntd.0011179 PMC10035853

[8] Lothigius A, Sjöling A, Svennerholm AM, Bölin I. Survival and gene expression of enterotoxigenic Escherichia coli during long-term incubation in sea water and freshwater. J Appl Microbiol. 2010;108(4):1441–9. 19804537 10.1111/j.1365-2672.2009.04548.x

[9] Lothigius A, Janzon A, Begum Y, Sjöling A, Qadri F, Svennerholm AM, Enterotoxigenic Escherichia coli is detectable in water samples from an endemic area by real-time PCR. J Appl Microbiol. 2008;104(4):1128–36. 17976169 10.1111/j.1365-2672.2007.03628.x

[10] Qadri F, Svennerholm AM, Faruque AS, Sack RB. Enterotoxigenic Escherichia coli in developing countries: epidemiology, microbiology, clinical features, treatment, and prevention. Clin Microbiol Rev. 2005;18(3):465–83. 16020685 10.1128/CMR.18.3.465-483.2005 PMC1195967

[11] Gonzales-Siles L, Sjöling Å. The different ecological niches of enterotoxigenic scherichia coli. Environ Microbiol. 2016;18(3):741–51. 26522129 10.1111/1462-2920.13106 PMC4982042

[12] Crofts AA, Giovanetti SM, Rubin EJ, Poly FM, Gutiérrez RL, Talaat KR, Enterotoxigenic E. coli virulence gene regulation in human infections. Proceedings of the National Academy of Sciences. 2018;115(38):E8968–E76. 10.1073/pnas.1808982115 PMC6156659 30126994

[13] Joffre E, Nicklasson M, Álvarez-Carretero S, Xiao X, Sun L, Nookaew I, The bile salt glycocholate induces global changes in gene and protein expression and activates virulence in enterotoxigenic Escherichia coli. Sci Rep. 2019;9(1):108. 30643184 10.1038/s41598-018-36414-z PMC6331568

[14] Gonzales L, Ali ZB, Nygren E, Wang Z, Karlsson S, Zhu B, Alkaline pH Is a signal for optimal production and secretion of the heat labile toxin, LT in enterotoxigenic Escherichia coli (ETEC). PLoS One. 2013;8(9):e74069. 24058516 10.1371/journal.pone.0074069 PMC3776858

[15] Joffré E, Xiao X, Correia MSP, Nookaew I, Sasse S, Globisch D, Analysis of Growth Phases of Enterotoxigenic Escherichia coli Reveals a Distinct Transition Phase before Entry into Early Stationary Phase with Shifts in Tryptophan, Fucose, and Putrescine Metabolism and Degradation of Neurotransmitter Precursors. Microbiol Spectr. 2022;10(4):e0175521. 35876501 10.1128/spectrum.01755-21 PMC9431495

[16] Joffré E, von Mentzer A, Svennerholm AM, Sjöling Å. Identification of new heat-stable (STa) enterotoxin allele variants produced by human enterotoxigenic Escherichia coli (ETEC). Int J Med Microbiol. 2016;306(7):586–94. 27350142 10.1016/j.ijmm.2016.05.016

[17] Haycocks JR, Sharma P, Stringer AM, Wade JT, Grainger DC. The molecular basis for control of ETEC enterotoxin expression in response to environment and host. PLoS Pathog. 2015;11(1):e1004605. 25569153 10.1371/journal.ppat.1004605 PMC4287617

[18] Abd El Ghany M, Barquist L, Clare S, Brandt C, Mayho M, Joffre E, Functional analysis of colonization factor antigen I positive enterotoxigenic Escherichia coli identifies genes implicated in survival in water and host colonization. Microb Genom. 2021;7(6). 10.1099/mgen.0.000554 PMC8461466 34110281

[19] Franchini AG, Egli T. Global gene expression in Escherichia coli K-12 during short-term and long-term adaptation to glucose-limited continuous culture conditions. Microbiology (Reading). 2006;152(Pt 7):2111–27. 16804185 10.1099/mic.0.28939-0

[20] Sarkhel R, Priyadarsini S, Mahawar M. Nutrient limitation and oxidative stress induce the promoter of acetate operon in Salmonella Typhimurium. Arch Microbiol. 2024;206(3):126. 38411730 10.1007/s00203-024-03863-2

[21] Kiupakis AK, Reitzer L. ArgR-independent induction and ArgR-dependent superinduction of the astCADBE operon in Escherichia coli. J Bacteriol. 2002;184(11):2940–50. 12003934 10.1128/JB.184.11.2940-2950.2002 PMC135064

[22] Cimdins A, Simm R. Semiquantitative Analysis of the Red, Dry, and Rough Colony Morphology of Salmonella enterica Serovar Typhimurium and Escherichia coli Using Congo Red. In: Sauer K, editor. c-di-GMP Signaling: Methods and Protocols. New York, NY: Springer New York; 2017. p. 225–41. 10.1007/978-1-4939-7240-1_18 28889298

[23] Costerton JW, Lewandowski Z, Caldwell DE, Korber DR, Lappin-Scott HM. MICROBIAL BIOFILMS. Annual Review of Microbiology. 1995;49(1):711–45. 10.1146/annurev.mi.49.100195.003431 8561477

[24] Gonzales-Siles L, Sjöling Å. The different ecological niches of enterotoxigenic Escherichia coli. Environ Microbiol. 2016;18(3):741–51. 26522129 10.1111/1462-2920.13106 PMC4982042

[25] Korhonen LK, Martikainen PJ. Comparison of the survival of Campylobacter jejuni and Campylobacter coli in culturable form in surface water. Canadian Journal of Microbiology. 1991;37(7):530–3. 1913357 10.1139/m91-089

[26] Pommepuy M, Fiksdal L, Gourmelon M, Melikechi H, Caprais MP, Cormier M, Effect of seawater on Escherichia coli beta-galactosidase activity. J Appl Bacteriol. 1996;81(2):174–80. 8760326 10.1111/j.1365-2672.1996.tb04496.x

[27] Flint KP. The long-term survival of Escherichia coli in river water. Journal of Applied Bacteriology. 1987;63(3):261–70. 3323155 10.1111/j.1365-2672.1987.tb04945.x

[28] Rozen Y, Belkin S. Survival of enteric bacteria in seawater. FEMS Microbiology Reviews. 2001;25(5):513–29. 11742689 10.1111/j.1574-6976.2001.tb00589.x

[29] Kingsley RA, Langridge G, Smith SE, Makendi C, Fookes M, Wileman TM, Functional analysis of Salmonella Typhi adaptation to survival in water. Environ Microbiol. 2018;20(11):4079–90. 30450829 10.1111/1462-2920.14458 PMC6282856

[30] Teteneva N, Sanches-Medeiros A, Sourjik V. Genome-wide screen of genetic determinants that govern Escherichia coli growth and persistence in lake water. The ISME Journal. 2024;18(1). 10.1093/ismejo/wrae096 PMC11188689 38874171

[31] Charlier D, Bervoets I. Regulation of arginine biosynthesis, catabolism and transport in Escherichia coli. Amino Acids. 2019;51(8):1103–27. 31267155 10.1007/s00726-019-02757-8

[32] Zhang YM, White SW, Rock CO. Inhibiting bacterial fatty acid synthesis. J Biol Chem. 2006;281(26):17541–4. 16648134 10.1074/jbc.R600004200

[33] Sah-Teli SK, Hynönen MJ, Sulu R, Dalwani S, Schmitz W, Wierenga RK, Insights into the stability and substrate specificity of the E. coli aerobic β-oxidation trifunctional enzyme complex. J Struct Biol. 2020;210(3):107494. 32171906 10.1016/j.jsb.2020.107494

[34] Yamanaka Y, Oshima T, Ishihama A, Yamamoto K. Characterization of the YdeO regulon in Escherichia coli. PLoS One. 2014;9(11):e111962. 25375160 10.1371/journal.pone.0111962 PMC4222967

[35] Ryan D, Pati NB, Ojha UK, Padhi C, Ray S, Jaiswal S, Global transcriptome and mutagenic analyses of the acid tolerance response of Salmonella enterica serovar Typhimurium. Appl Environ Microbiol. 2015;81(23):8054–65. 26386064 10.1128/AEM.02172-15 PMC4651094

[36] Gibbons E, Tamanna M, Cherayil BJ. The rpoS gene confers resistance to low osmolarity conditions in Salmonella enterica serovar Typhi. PLoS One. 2022;17(12):e0279372. 36525423 10.1371/journal.pone.0279372 PMC9757558

[37] Zhang XS, García-Contreras R, Wood TK. Escherichia coli transcription factor YncC (McbR) regulates colanic acid and biofilm formation by repressing expression of periplasmic protein YbiM (McbA). Isme j. 2008;2(6):615–31. 18309357 10.1038/ismej.2008.24

[38] Romantsov T, Guan Z, Wood JM. Cardiolipin and the osmotic stress responses of bacteria. Biochim Biophys Acta. 2009;1788(10):2092–100. 19539601 10.1016/j.bbamem.2009.06.010 PMC3622477

[39] Huang KC, Mukhopadhyay R, Wen B, Gitai Z, Wingreen NS. Cell shape and cell-wall organization in Gram-negative bacteria. Proceedings of the National Academy of Sciences. 2008;105(49):19282–7. 10.1073/pnas.0805309105 PMC2592989 19050072

[40] Baron S, Leulmi Z, Villard C, Olaitan AO, Telke AA, Rolain JM. Inactivation of the arn operon and loss of aminoarabinose on lipopolysaccharide as the cause of susceptibility to colistin in an atypical clinical isolate of proteus vulgaris. Int J Antimicrob Agents. 2018;51(3):450–7. 29203405 10.1016/j.ijantimicag.2017.11.017

[41] Breazeale SD, Ribeiro AA, McClerren AL, Raetz CR. A formyltransferase required for polymyxin resistance in Escherichia coli and the modification of lipid A with 4-Amino-4-deoxy-L-arabinose. Identification and function oF UDP-4-deoxy-4-formamido-L-arabinose. J Biol Chem. 2005;280(14):14154–67. 15695810 10.1074/jbc.M414265200

[42] Ezadi F, Ardebili A, Mirnejad R. Antimicrobial Susceptibility Testing for Polymyxins: Challenges, Issues, and Recommendations. Journal of Clinical Microbiology. 2019;57(4):10.1128/jcm.01390-18. PMC6440778 30541939

[43] Simpson BW, Trent MS. Pushing the envelope: LPS modifications and their consequences. Nat Rev Microbiol. 2019;17(7):403–16. 31142822 10.1038/s41579-019-0201-x PMC6913091

[44] Gallardo A, Iglesias MR, Ugarte-Ruiz M, Hernández M, Miguela-Villoldo P, Gutiérrez G, Plasmid-mediated Kluyvera-like arnBCADTEF operon confers colistin (hetero)resistance to Escherichia coli. Antimicrob Agents Chemother. 2023;65(5). 10.1128/AAC.00091-21 PMC8092862 33685891

[45] Hwang D, Kim SM, Kim HJ. Transcriptome changes and polymyxin resistance of acid-adapted Escherichia coli O157:H7 ATCC 43889. Gut Pathog. 2020;12(1):52. 33292490 10.1186/s13099-020-00390-5 PMC7709258

[46] Ryan D, Pati NB, Ojha UK, Padhi C, Ray S, Jaiswal S, Global Transcriptome and Mutagenic Analyses of the Acid Tolerance Response of Salmonella enterica Serovar Typhimurium. Applied and Environmental Microbiology. 2015;81(23):8054–65. 26386064 10.1128/AEM.02172-15 PMC4651094

[47] Prasad SV, Fiedoruk K, Zakrzewska M, Savage PB, Bucki R. Glyoxylate Shunt and Pyruvate-to-Acetoin Shift Are Specific Stress Responses Induced by Colistin and Ceragenin CSA-13 in Enterobacter hormaechei ST89. Microbiol Spectr. 2023;11(4):e0121523. 37338344 10.1128/spectrum.01215-23 PMC10434160

[48] Mhaya A, Bégu D, Tounsi S, Arpin C. MgrB Inactivation Is Responsible for Acquired Resistance to Colistin in Enterobacter hormaechei subsp. steigerwaltii. Antimicrob Agents Chemother. 2020;64(6). 10.1128/AAC.00128-20 PMC7269514 32253218

[49] Sharma E, Kelso C, Zhang S, Guo Y, Sivakumar M, Jiang G. Stability of Colistin and Carbapenems in Water and Wastewater. ACS ES&T Water. 2023;3(11):3496–504.

[50] von Mentzer A, Connor TR, Wieler LH, Semmler T, Iguchi A, Thomson NR, Identification of enterotoxigenic Escherichia coli (ETEC) clades with long-term global distribution. Nat Genet. 2014;46(12):1321–6. 25383970 10.1038/ng.3145

[51] von Mentzer A, Blackwell GA, Pickard D, Boinett CJ, Joffré E, Page AJ, Long-read-sequenced reference genomes of the seven major lineages of enterotoxigenic Escherichia coli (ETEC) circulating in modern time. Scientific Reports. 2021;11(1):9256. 33927221 10.1038/s41598-021-88316-2 PMC8085198

[52] Sonika S, Singh S, Mishra S, Verma S. Toxin-antitoxin systems in bacterial pathogenesis. Heliyon. 2023;9(4):e14220. 37101643 10.1016/j.heliyon.2023.e14220 PMC10123168

[53] Liu F, Yang X, Wang Z, Nicklasson M, Qadri F, Yi Y, Draft genomes of four enterotoxigenic Escherichia coli (ETEC) clinical isolates from China and Bangladesh. Gut Pathog. 2015;7:10. 25932050 10.1186/s13099-015-0059-z PMC4415261

[54] Evans DJ Jr., Evans DG. Three characteristics associated with enterotoxigenic Escherichia coli isolated from man. Infect Immun. 1973;8(3):322–8. 4581006 10.1128/iai.8.3.322-328.1973 PMC422851

[55] Crossman LC, Chaudhuri RR, Beatson SA, Wells TJ, Desvaux M, Cunningham AF, A Commensal Gone Bad: Complete Genome Sequence of the Prototypical Enterotoxigenic Escherichia coli Strain H10407. Journal of Bacteriology. 2010;192(21):5822–31. 20802035 10.1128/JB.00710-10 PMC2953697

[56] Sjöling A, Wiklund G, Savarino SJ, Cohen DI, Svennerholm AM. Comparative analyses of phenotypic and genotypic methods for detection of enterotoxigenic Escherichia coli toxins and colonization factors. J Clin Microbiol. 2007;45(10):3295–301. 17687011 10.1128/JCM.00471-07 PMC2045327

[57] Langmead B, Trapnell C, Pop M, Salzberg SL. Ultrafast and memory-efficient alignment of short DNA sequences to the human genome. Genome Biol. 2009;10(3):R25. 19261174 10.1186/gb-2009-10-3-r25 PMC2690996

[58] Anders S, Pyl PT, Huber W. HTSeq—a Python framework to work with high-throughput sequencing data. Bioinformatics. 2014;31(2):166–9. 25260700 10.1093/bioinformatics/btu638 PMC4287950

[59] Love MI, Anders S, Kim V, Huber W. RNA-Seq workflow: gene-level exploratory analysis and differential expression. F1000Res. 2015;4:1070. 26674615 10.12688/f1000research.7035.1 PMC4670015

[60] Liberzon A. A description of the Molecular Signatures Database (MSigDB) Web site. Methods Mol Biol. 2014;1150:153–60. 24743996 10.1007/978-1-4939-0512-6_9

